# IL-33/Vitamin D Crosstalk in Psoriasis-Associated Osteoporosis

**DOI:** 10.3389/fimmu.2020.604055

**Published:** 2021-01-08

**Authors:** Massimo De Martinis, Lia Ginaldi, Maria Maddalena Sirufo, Enrica Maria Bassino, Francesca De Pietro, Giovanni Pioggia, Sebastiano Gangemi

**Affiliations:** ^1^ Department of Life, Health and Environmental Sciences, University of L’Aquila, L’Aquila, Italy; ^2^ Institute for Biomedical Research and Innovation (IRIB), National Research Council of Italy (CNR), Messina, Italy; ^3^ School and Operative Unit of Allergy and Clinical Immunology, Department of Clinical and Experimental Medicine, University of Messina, Messina, Italy

**Keywords:** osteoporosis, psoriasis, IL-33, vitamin D, osteoimmunology, skin, bone, cytokines

## Abstract

Patients with psoriasis (Pso) and, in particular, psoriatic arthritis (PsoA) have an increased risk of developing osteoporosis (OP). It has been shown that OP is among the more common pathologies associated with Pso, mainly due to the well-known osteopenizing conditions coexisting in these patients. Pso and OP share common risk factors, such as vitamin D deficiency and chronic inflammation. Interestingly, the interleukin (IL)-33/ST2 axis, together with vitamin D, is closely related to both Pso and OP. Vitamin D and the IL-33/ST2 signaling pathways are closely involved in bone remodeling, as well as in skin barrier pathophysiology. The production of anti-osteoclastogenic cytokines, e.g., IL-4 and IL-10, is promoted by IL-33 and vitamin D, which are stimulators of both regulatory and Th2 cells. IL-33, together with other Th2 cytokines, shifts osteoclast precursor differentiation towards macrophage and dendritic cells and inhibits receptor activator of nuclear factor kappa-B ligand (RANKL)-induced osteoclastogenesis by regulating the expression of anti-osteoclastic genes. However, while the vitamin D protective functions in OP and Pso have been definitively ascertained, the overall effect of IL-33 on bone and skin homeostasis, because of its pleiotropic action, is still controversial. Emerging evidence suggests a functional link between vitamin D and the IL-33/ST2 axis, which acts through hormonal influences and immune-mediated effects, as well as cellular and metabolic functions. Based on the actions of vitamin D and IL-33 in Pso and OP, here, we hypothesize the role of their crosstalk in the pathogenesis of both these pathologies.

## Introduction

Psoriasis (Pso) is a chronic autoimmune multifactorial disease that is associated with systemic inflammation. It presents with skin erythematous plaques, covered by characteristic white silvery scales (1). It is characterized by increased proliferation of keratinocytes, perivascular skin infiltration by cells belonging to both the adaptive and innate immune system, and imbalances in apoptotic and autophagic pathways (2).

Through the production of inflammatory cytokines, activated and autoreactive immune cells play central roles in its pathogenesis (3). In both skin and blood of psoriatic patients, there are increased levels of various cytokines, growth factors, and chemokines (4). Patients suffering from Pso, particularly those with psoriatic arthritis (PsoA) or more severe forms of the disease, develop multiple comorbidities in addition to joint diseases, including cardiovascular and rheumatologic disorders, infections, obesity, and diabetes (5–7). The link between these comorbidities is likely systemic inflammation (8). Recently, osteoporosis (OP) is also considered to be a relevant comorbidity in Pso (9). Therefore, patients with Pso are now recognized to be at increased risk of pathologic fractures and OP, so today, it is commonly believed that Pso patients might benefit from increased screening for OP (10).

OP is defined as a generalized disease of the skeleton, characterized by low bone mineral density (BMD) and altered microarchitecture, leading to increased bone fragility and, as a result, increased risk of fractures (11, 12). In addition to senile and postmenopausal OP, secondary OP may also occur as a consequence of various pathologies, including endocrinopathies, rheumatic and neoplastic diseases, malnutrition, chronic inflammatory conditions, and 1,25-dihydroxyvitamin D (vitamin D) deficiency (13–17). Several potential mechanisms may explain the association between Pso and OP, including a low vitamin D level, chronic inflammation, and drug usage (18–20). In particular, proinflammatory cytokines, such as interleukin (IL)-1, IL-6, IL-11, IL-15, IL-17, and tumor necrosis factor (TNF)-alpha, might accelerate bone loss, whereas other cytokines, mostly of the Th2 profile, e.g., IL-4 and IL-33, are usually considered osteoprotective (21).

IL-33 and vitamin D are emerging pathogenetic factors of both Pso and OP. However, their role in the development of these associated pathologies is complex and not yet fully clarified. Here, we hypothesize a mechanistic link between vitamin D and IL-33 in patients with Pso and associated OP.

## Pathogenic Mechanisms Linking Pso and OP

Several pathogenetic mechanisms link Pso and OP (**Box 1**). Although there are conflicting results in the literature about this association and its pathogenetic mechanisms, the majority of studies describe a decreased BMD in patients with long-term Pso and PsoA (9, 22, 23). Like Pso, OP can also be considered a systemic pathology (21). Although it is mainly linked to menopause and aging (14, 24), OP can also accompany a wide range of pathologies, in particular, those with an important inflammatory substrate, including dermatological disorders (25–28).

Many cytokines are involved in the regulation of bone turnover, and most of them also underlie the inflammatory background of Pso (4, 21, 29). Vitamin D deficiency is among the main risk factors of both pathologies (29–31). The hypothesized mechanisms underlying the potential association between Pso and OP involves enhanced bone resorption secondary to increased concentrations of osteoclastogenic cytokines, such as TNF-alpha, IL-6, IL-12, IL-23, or IL-17 (31, 32). The central signal pathway in bone resorption is the system of the receptor activator of nuclear factor kappa-B (NF-kB) ligand (RANKL), mainly expressed by osteoblasts, that binds to its receptor RANK on the osteoclast precursor cells, inducing their differentiation into mature osteoclasts and thus leading to bone resorption. Osteoprotegerin (OPG), the decoy receptor of RANKL, prevents bone resorption by inhibiting osteoclastogenesis. In the pathogenesis of OP, the RANKL-RANK-OPG axis is unbalanced (12). Inflammatory cytokines, whose production is increased in Pso, exert osteoclastogenic effects mainly through the enhancement of RANKL expression. In particular, IL-17, produced by T helper type 17 (Th17) cells, plays a pivotal role in the bone loss of inflammatory conditions, including Pso, by enhancing RANKL expression on osteoblasts and synovial fibroblasts. Moreover, IL-17 stimulates the production of other inflammatory and osteoclastogenic cytokines, such as TNF-α, IL-1, and IL-6, which accelerate osteoclastogenesis, further facilitating the development of OP. High surface expression of RANKL on Th17 cells characterizes the so-called osteoclast subsets of T lymphocytes, that strongly enhance bone resorption. On the contrary, T regulatory (Treg) cells inhibit osteoclastogenesis and support bone formation. Therefore, while Th17 cells induce osteoclastogenesis, mainly by secreting IL-17, IL-4 enhanced Treg exert anti-osteoclastogenic activity by producing suppressor cytokines, including IL-10, and transforming growth factor-beta (TGF-β) (21). An impaired Th17/Treg cell balance is central in the inflammatory background of both Pso and OP. Treg cells are responsible for the maintenance of self-tolerance, thus inhibiting autoimmune diseases, including Pso, and are also able to suppress RANKL‐induced osteoclastogenesis, whereas pro-inflammatory Th17 cells contribute to the induction and propagation of inflammation. Th17 cells, converted from Foxp3+ Treg in inflamed tissues, such as psoriatic skin lesions, comprise the most potent osteoclastogenic T cell subset in inflammatory bone loss (33). In the complex cytokine network involved in Pso, a crucial role is also exerted by IL‐12/Th1 and IL‐23/Th17 axis, by linking components of adaptive and innate immunity in an inflammatory crosstalk. In the skin, activated dendritic cells (DCs) trigger Th1 and Th17 cells to differentiate and release IFN-γ and TNF-α, and IL-17 and IL-22, respectively, which promote keratinocyte proliferation. IL-23 is a heterodimeric cytokine composed of two subunits, p19 and p40. The latter subunit is shared by the Th1-inducing cytokine IL-12. Upon skin injury, IL-23 produced by activated DCs, stressed keratinocytes and other non-immune cells directly drives expansion and survival of Th17 lymphocytes, stimulates IL-17 production, and induces downregulation of IL‐10, involved in Treg cell function, thus creating a self-amplifying inflammatory response that drives the development of skin lesions infiltrated with a mixture of inflammatory cell populations. Innate lymphoid cells (ILCs) represent a heterogeneous group of immune cells lacking specific antigen receptors or T/B cell markers. ILC3, which express the transcription factor retinoid‐related orphan receptor γt (ROR γt) and are characterized by the ability to produce Th17 and IL-22 cytokines, are increased in Pso. ILC3 constitutively express the IL-23 receptor, thus representing a target for IL-23-mediated IL-17 and IL-22 increased production. The secretion of IL-17 and IL-22 from IL-23-stimulated ILC3 promotes the aberrant keratinocyte differentiation and hyperproliferation, typically observed in Pso (34). IL-12 and IL-23 involved in skin inflammation, are also critical to inflammation-induced bone resorption, *via* a number of direct and indirect effects that modulate osteoclast formation. In the bone, IL-23 upregulates RANK on preosteoclasts and induces Th17 cells to produce IL-17, which acts on osteoblasts to secrete RANKL. Th17 cells also secrete RANKL directly and further induce osteoclast formation and secretion of bone-degrading enzymes leading to bone destruction (35). Many other cytokines involved in bone remodeling have recently been shown to also exert roles in the pathogenesis of Pso, including, in particular, IL-33 (36–39). In addition, some treatments used in Pso might contribute to bone loss, for example, corticosteroids and cyclosporin, particularly when used systemically (18–20), whereas treatments at the systemic level aimed at reducing inflammation, e.g., biologics or methotrexate, could reduce the risk associated with osteoporotic fractures (40). Finally, psoriatic patients, especially those with associated inflammatory arthritis, engage less in physical activities and tend to cover affected body surfaces with consequent decreases in osteoformation and vitamin D synthesis (41, 42). In particular, the lack of sun exposure, which may affect mainly psoriatic patients with extensive skin involvement and/or arthritis through vitamin D deficiency, negatively affects calcium metabolism, further increasing bone resorption and leading to the onset of OP (43). Psoriatic patients of both sexes appear to have a high prevalence of OP and vitamin D deficiency (10, 44). Low levels of vitamin D metabolizing enzymes (CYP27A1 and CYP27B1) within psoriatic lesions have also been documented (9, 45), and vitamin-D treatment in psoriatic patients has been associated with clinical improvement of skin lesions (46, 47).

Pso is related to vitamin D deficiency through both inflammation and a lack of sun exposure (48). Interestingly, vitamin D deficiency and OP are frequently recognized in the great majority of associated Pso comorbidities (21, 49) For example, Pso is frequently associated with metabolic syndrome, increased body mass index, and obesity (50). All of these associated conditions are characterized by both an inflammatory background and increased fat deposits, in which vitamin D tends to accumulate because of its liposolubility, consequently reducing its circulating bioavailable levels. In these patients, vitamin D deficiency is commonly related to hyperglycemia and higher levels of cholesterol, low-density lipoprotein, and triglycerides (51, 52). Therefore, despite the tendency for a higher body mass index in patients with Pso, which might have a protective effect against OP, both vitamin D deficiency and systemic inflammation can still induce BMD loss.

Chronic inflammation itself has been related to low vitamin D levels and decreased BMD (53, 54). Under pathological conditions, the equilibrium between bone formation and resorption, which physiologically ensures skeletal homeostasis, is shifted towards osteoclast-mediated bone resorption. Proinflammatory cytokines, such as TNF-alpha and IL-17, are notoriously associated with osteoclastic bone resorption in inflammatory diseases (21). These cytokines trigger osteoclastogenesis through the activation of a series of transcription factors, such as NF-kappa B. In PsoA, synovial inflammation can further facilitate the onset of local and systemic OP (29). In these patients, an increase in osteoclast progenitors (OCP) correlates with the extent of joint erosions and inflammation markers (41).

The association between Pso and OP is therefore supported by the existence of pathophysiological mechanisms, namely, excessive production of proinflammatory cytokines that are able to activate osteoclastogenesis, and the frequent lack of vitamin D characterizing psoriatic patients (55, 56).

However, studies on OP development and the increased risk of fractures in patients with Pso are still somewhat controversial, because of the complexity of the network of interconnected cytokines and the regulatory factors linking the two pathologies.

Box 1 **Main pathogenetic mechanisms in Pso and OP.**

Key points:

Vitamin D deficiency

Overexpression of IL-33/IL-31 axis

Chronic systemic inflammation

Increased production of inflammatory and osteoclastogenic cytokines

Unbalanced RANKL-RANK-OPG signaling pathway

Overexpression of IL-23/IL-17 axis

Impaired Th17/Treg cell balance

Increased secretion of IL-17 and IL-22 from IL-23-stimulated ILC3

## The Role of Vitamin D

Vitamin D has multiple functions, including hormonal and immunological control (**Box 2**). Vitamin D regulates more than 200 genes involved in cell proliferation and differentiation, the secretion of different hormones, and immune cell activity (54, 57). The lack of vitamin D in Pso is widely recognized as an important factor that contributes to the development of OP (58). Vitamin D regulates calcium and phosphorous metabolism and parathyroid hormone (PTH) secretion and function. For these activities, it has important implications for the maintenance of skeletal integrity. As a consequence of vitamin D deficiency, bone mineralization disorders arise, mainly through an imbalance in the calcium/phosphorus ratio. Vitamin D is indispensable for physiological bone turnover. In particular, in order to prevent OP, supplementation with vitamin D is strongly recommended as a support for anti-osteoporotic therapies (12, 15, 53). Moreover, in addition to its effects on bone, vitamin D also exerts important functions in skin homeostasis and its deficiency is linked with Pso development (48, 59). Vitamin D is therefore no longer considered to just be an essential factor for the maintenance of a normal skeletal structure, but its extraskeletal effects, including cell cycle regulation and immune modulation, are becoming increasingly known (60–62).

The epidermis is the natural source of vitamin D synthesis through the action of ultraviolet light (53), and in turn, vitamin D functions as a key regulator of cutaneous barrier homeostasis. The skin therefore acts as the site of vitamin D synthesis and also as the target organ for its biologically active form (47). Keratinocytes contain enzymes needed for the production of the active form of vitamin D, 1,25 (OH)2D, and express its receptor, vitamin D receptor (VDR), thus being able to synthesize and also respond to vitamin D. Through this signaling pathway, vitamin D is involved in regulating epidermal development, keratinocyte proliferation, differentiation and apoptosis (54), and the synthesis of keratins, involucrin, transglutaminase, loricrin, and filaggrin, helping to modulate skin barrier function (63, 64). These control mechanisms are partly due to its ability to increase intracellular calcium by inducing the production of phospholipases and calcium receptors needed for calcium-dependent keratinocyte differentiation. After activation, VDRs interact with retinoid X receptor (RXR) to exert their functions. VDRs, as well as enzymes able to synthesize the active form of the vitamin, namely 1,25-dihydroxy-vitamin D, are expressed in several tissues beyond the kidneys and bones, including the skin and the immune system, suggesting that vitamin D is involved in many other functions besides the metabolic ones (53, 65). An association between Pso susceptibility and VDR polymorphisms, as well as between reduced tight-junction proteins and decreased VDR expression in psoriatic skin, has been described (57). It has been shown that vitamin D also exerts central roles in both humoral and cellular regulation by suppressing T-cell proliferation and Th2 cell development and through the induction of regulatory T cells, cytokine production modulation, and dendritic cell regulation (66–69).

Through the downregulation of IL-12 production, vitamin D suppresses the maturation of Th1 cells, leading to increased Th2 lymphocyte proliferation (47, 70). However, vitamin D also promotes regulatory T cell maturation and increases IL-10 synthesis, exerting inhibitory effects on Th2 immune responses (71).

Moreover, vitamin D inhibits proliferation and induces apoptosis in various cell types (54). Following dysregulation of the cell cycle, autophagy and apoptosis have important roles in inflammatory processes (72) underlying both OP and Pso. This could represent a further mechanism through which vitamin D deficiency contributes to their pathogeneses (73–76). In psoriatic skin inflammation, cytokines and chemokines produced by dysregulated T lymphocytes play key roles (77). As in Pso, in the pathogenesis of OP, these immune-mediated mechanisms are strongly involved (21). By inhibiting the production of Th1 and Th17 cytokines and stimulating T cells to secrete anti-inflammatory Th2 cytokines in both the skin and bone immune systems, vitamin D reduces the production of osteoclastogenic cytokines as well as the psoriatic inflammatory process (64, 78, 79). Moreover, since vitamin D promotes Th2 and Treg differentiation rather than Th1 and Th17 proinflammatory lymphocytes (48) and inhibits B cell differentiation, thus interfering with the production of antibodies by plasma cells (70), its deficiency is also associated with an increased risk of developing autoimmune diseases (80–82), including Pso, which is a Th1-Th17-Th22-based inflammatory disease involving innate and acquired immunity (53). Therefore, despite being historically associated with rickets, osteomalacia, and OP, now considered an inflammatory Th1-mediated disease (83), vitamin D deficiency has recently been recognized as a risk factor for chronic systemic diseases, including diabetes, neoplasia, allergies, infections, autoimmune, cardiovascular, and neurodegenerative diseases (76). Pso is histologically characterized by keratinocyte hyperproliferation, derangement of the epidermal barrier function, and infiltration of the skin by multiple activated inflammatory cells (53). As a key modulator of systemic inflammation, vitamin D normalizes the dysregulated distribution of CD26 and ICAM-1 integrins in the dermal–epidermal junction of psoriatic skin and suppresses the inflammatory profile of monocytes/macrophages (55), down-regulating the production of cytokines, such as IL-1β, IL-6, IL-8, and TNF-alpha (48, 80). All of these cytokines are involved in inflammatory processes leading to Pso and OP (47, 83, 84). Indeed, vitamin D and its analogues suppress the proliferation of keratinocytes and their proinflammatory molecule production, therefore improving psoriatic lesions and contributing to skeletal health by suppressing bone resorption and favoring bone formation.

Box 2 **Vitamin D functions.**

Key points:

Calcium/phosphorous metabolism regulation

Parathyroid hormone secretion and function control

Regulation of cutaneous barrier homeostasis

Keratinocyte proliferation, apoptosis and function control

Th1/Th2 cell development modulation

Induction of regulatory T cells

Down-regulation of Th17 cells

Down-regulation of nflammatory cytokine production

## The Role of IL-33

IL-33 is a cytokine that can promote Th2 response, but also has broad activities (**Box 3**) including the promotion of activated Th1 and CD8+ cytotoxic cells and also Treg cells that express ST2 receptor (85, 86). As well as vitamin D, also IL-33 is involved in various biological processes, including tissue homeostasis and repair, cell proliferation, and the immune response, and it plays key roles in the pathogenetic mechanisms of several diseases, such as allergic, autoimmune, neoplastic, and cardiovascular diseases. In particular, IL-33 is involved in skin diseases, including Pso (39, 87, 88), and in OP through its role in bone remodeling (89–92). Its receptor complex consists of a primary receptor, ST2, and an accessory IL-1 receptor protein. The ST2 receptor exists in two different forms: a transmembrane isoform acts as a cellular receptor, whereas a soluble form (sST2) plays the role of decoy receptor inhibiting IL-33 activity (93, 94). Interestingly, the IL-33/ST2 axis is involved in both Th2 and Th1/Th17 responses (88) as well as in the activation of Treg and natural killer (NKT) cells, B and NK lymphocytes, neutrophils, and macrophages (91, 95–98). A regulatory feedback axis exists between stromal cells expressing IL-33 and adipose resident ST2+ Tregs (99, 100). Obesity alters this homeostatic cellular network and promotes the inflammatory response (101, 102). Several immune cells involved in the type 2 immune response express ST2. Among others, examples of such immune cells are the group 2 innate lymphoid cells (ILC2), eosinophils, basophils, and DCs, as well as mast cells and Th2 cells (103). Increased amphiregulin (AREG) levels are associated with vitamin D deficiency. The ILC2-associated marker AREG, whose encoding gene is a target of vitamin D, activates IL-33-responsive ST2 T cells and ILC2s (104), which function as key effectors producing Th2 cytokines, including IL-4, IL-5, IL-9, and IL-13 (105), when released from necrotic and/or apoptotic epithelial cells. On the other hand, TNF-alpha, INF-gamma, and IL-17, major effectors of the Th1/Th17 responses in Pso and OP pathogenesis, stimulate the release of IL-33. Both skin lesions and unaffected skin biopsies of Pso patients (36, 37, 95) express high IL-33 levels (103, 106). The rapid IL-33 release from keratinocytes after skin injury could be a crucial mechanism involved in the pathogenesis of Pso (107–110). Once released in the local microenvironment, IL-33 could activate mast cells and neutrophils as well as Th1/Th17 cells, triggering both innate and adaptive immune responses. Mast cells can activate other innate immune cells, such as eosinophils and neutrophils, and, in turn, can recruit and activate keratinocytes. The above-mentioned interactions are pivotal for the emergence of skin inflammation and Pso lesions (111). Both TNF-alpha and INF-gamma in psoriatic skin increase the expression of IL-33 which, in turn, is able to suppress the actions of other cytokines (38). Also, IL-17 upregulates IL-33 expression in normal human epidermal keratinocyte (NHEK) cultures through the activation of the p38/MAPK, ERK, and JAK/STAT pathways (112). IL-33 reinforces the TNF-alpha induced secretion of IL-6, VEGF, and MCP-1. However, data on IL-33 serum levels in Pso are controversial (113). A large number of studies suggest that a localized, rather than a generalized, IL-33 linked inflammatory pattern is evident in Pso (114). IL-33 serum levels are not always increased in the serum of patients with Pso, notwithstanding the increased levels of this cytokine in inflamed skin (103, 111, 114). It has also been suggested that IL-33 expression in the nuclei of keratinocytes following IL-17 stimulation may represent a regulatory mechanism aimed at attenuating immune reactions (110).

The expression levels of both IL-33 and ST2 are up-regulated in psoriasis, likely as a consequence of keratinocyte damage. IL-33/ST2 signals may subsequently trigger the activation of neutrophils and mast cells, leading to Pso development. This pathogenetic hypothesis assumes interplay between keratinocytes and the immune system (88, 107, 108). IL-33 exerts a dual function. It acts as a cytokine or as a nuclear factor when involved extracellularly or intracellularly, respectively, thus participating in both inflammation processes and gene expression regulation (4). Upon cellular stress and tissue damage, released IL-33 functions as an alarmin and activates innate and adaptive immune cells, inflammation, or tissue repair. IL-33 is considered a key alarmin in both Pso and OP, although it exerts contrasting effects in these pathological conditions (115). Continuous alarmin release promotes polarization toward a Th1 phenotype, and this effect contributes to the initial background of the local hyperinflammatory environment that characterizes Pso (92, 107).

The IL-33/ST2 pathway intervenes in the pathogenesis of Th2-related diseases, such as allergies (103), but could also exert some protective effects in other inflammatory pathologies, such as cardiovascular diseases and OP, mainly depending on genetics, disease duration, and the cytokine microenvironment (116). In particular, the role of IL-33 in OP and Pso is still debated. Some authors have reported IL-33 inhibition of osteoclast formation (117), whereas others described an IL-33-induced reduction in osteoprotegerin expression by osteoblasts and an increase in osteoclastogenic factor release (118), thus suggesting an IL-33 mediated bone resorption induction during inflammation (98). IL-33 stimulates mast cells to produce IL-6 and IL-13 *via* the canonical NF-kB signaling and p38 pathways. The MAPK-activated protein kinases MK2 and MK3, function as sensors of cell injury and exert pivotal roles in IL-33-induced cytokine production by mast cells in inflammatory responses (87, 98, 107). Due to its pleiotropic nature, IL-33 exerts contrasting roles in different diseases (87, 88), i.e., IL-33 can either drive the underlying inflammation or promote its resolution (99, 113) according to the individual inflammatory context. Disease severity and changes in relation to hormonal influences contribute to the bone remodeling effects of IL-33. For example, notwithstanding the decreased serum IL-33 levels in women undergoing menopause with OP, its protective effect on the bone disappears as OP progresses, likely due to the interference of other proinflammatory osteoclastogenic cytokines. In particular, IL-33 has controversial roles in bone remodeling (115, 119, 120). The majority of studies suggest prevalent anti-osteoclastogenic and osteoanabolic functions of IL-33, but the reduced expression of OPG by osteoblasts and the induced production of the osteoclastogenic cytokine IL-31 (97), have also been reported, suggesting that in particular inflammatory conditions, including arthritis and Pso, IL-33 can induce bone resorption. This hypothesis seems to be supported by the finding of periarticular bone erosions and systemic OP in PsoA, in which IL-33 plays a pathogenetically important role.

Box 3 **IL-33 functions.**

Key points:

Induction of Th2 responses and Th2 cytokine production

Promotion of activated Th1 and CD8+ cytotoxic cells

Activation of Treg cells expressing ST2 receptor

Gene expression regulation

Activation of inflammation and tissue ripair upon danger signals

Mast cell and neutrophil activation in inflamed skin

Enhancement of TNFα induced secretion of IL-6, VEGF, and MCP-1

## The Relationship Between Vitamin D and the IL-33/ST2 Axis in Psoriasis-Associated Osteoporosis

### Link Between Vitamin D and IL-33

There is a close relationship between vitamin D and the IL-33/ST2 axis in bone and skin homeostasis. Nonetheless, the exact role of vitamin D in IL-33 activities, which creates vicious circles in the pathogenesis of Pso-associated OP, remains controversial (91, 93). It is likely that vitamin D and IL-33 share some signal pathways and need each other to perform some important immunological and metabolic functions both the skin and bone levels. In particular, there is evidence that, in some biological processes, they act in synergy, while in other cases, they act by controlling and modulating each other (71). Here, we hypothesize a mechanistic link between vitamin D and IL-33 in patients with Pso and associated OP.

### Vitamin D and IL-33 Immunological Crosstalk

IL-33 shares many of the immunoregulatory effects of vitamin D, either potentiating or modulating them (**Box 4**). IL-33 is produced by cells regulated by vitamin D, and often their tissue targets and signal pathways are shared. For example, both vitamin D and IL-33 promote the differentiation of Th2 lymphocytes by inhibiting Th1 differentiation and also act as inducers of immunoregulatory cells. Vitamin D can downregulate the production of inflammatory cytokines and chemokines (121). Moreover, vitamin D activity is determined through vitamin D receptors, which are present not only in the skeleton but also in different types of cells, including antigen-presenting-cells, immune cells, and keratinocytes. In particular, DCs, lymphocytes, monocytes/macrophages, neutrophils, and epithelial cells can produce IL-33 and other cytokines involved in both bone remodeling and Pso inflammation, including IL-31, IL-17, and TNF-alpha (29, 62, 107). IL-33 therefore orchestrates the immune cascade of Pso. Moreover, it takes part in the bone remodeling process (115). Vitamin D plays a central role in both bone turnover and immune regulation. In addition, vitamin D is also implicated in skin homeostasis, acting as a potent immune system modulator and suppressing dendritic cell maturation. Serum vitamin D levels are decreased in Pso patients and, in particular, in those with associated OP.

The impact of low vitamin D levels has been widely investigated in both Pso and OP, as has the role of IL-33. Th2-related cytokines, including IL-4, IL-31, and IL-33, have recently been shown to play important roles in bone remodeling as well as in Pso skin inflammation and PsoA (47, 65). IL-33 may act as an alarmin, exerting both repairing and damaging processes and functioning as a nuclear transcription factor. Similarly, the vitamin D receptor, functioning as a ligand-activated transcription factor, regulates the activation or repression of gene transcription (121). Moreover, vitamin D deficiency likely independently contributes to the increased incidence of OP in Pso patients.

The relationship between IL-33 and vitamin D is highly complex and extremely variable. For example, IL-33 contributes to inflammatory reactions involving vitamin D deficiency but could also counteract some of its deleterious effects, mainly depending on the clinical context as well as on the cytokine and hormonal milieu. As a consequence, it is conceivable that although both vitamin D and IL-33 in the bone environment exert protective effects against OP, in inflamed Pso skin and in PsoA, the deleterious effects of vitamin D deficiency and IL-33/ST2 axis activation potentiate each other (85, 100). Decreased vitamin D and increased IL-33 levels are also both associated to Th2 immunity in allergic inflammatory diseases (122), suggesting that they play contrasting roles in allergies (71). Based on these considerations, a potential complex interaction between vitamin D and IL-33 has been hypothesized, not only in allergic diseases but also in other clinical conditions in which their roles have also been demonstrated, including Pso and OP. Vitamin D promotes anti-inflammatory IL-10 synthesis by inducing α-1-antitrypsin expression in CD4+ T cells (123). IL-10, as an immunosuppressive cytokine, directly limits Th2 cell differentiation and survival during allergic airway inflammation (124). On the other hand, IL-33 induces Th2 cytokines, including IL-31, thus exerting a pivotal role in orchestrating the recruitment and activation of effector cells of the allergic response. IL-31 is a cytokine produced by CD4+ T cells which has a potent immunological link with IL-33 and plays important roles in allergic inflammation and atopic dermatitis as well as in Pso (38, 88). A similar close link has been highlighted as being an important factor in the alteration of the bone remodeling process that underlies OP (92). The IL-31 receptor IL-31RA, the oncostatin M receptor, and the IL-33 receptor ST2 are related to the immunopathological mechanisms of Pso and OP. The ST2 receptor of IL-33, which is a critical component of Th2 responses, stimulates the production of IL-31 (88). At the same time, sST2, which is increased in Pso patients and acts as a decoy receptor for IL-33, is a negative modulator of the IL-33/ST2 axis. There is evidence that a perturbation of this axis exerts essential roles in OP (91). Plasma sST2 levels were found to be correlated with decreased cortical BMD and deterioration of the bone microstructure in Pso. Vitamin D enhances the synthesis of the IL-33 inhibitor sST2, counteracting inflammation in psoriatic skin (66). The association between increased sST2 and decreased vitamin D in Pso might synergistically contribute to the effect of inflammatory mediators in inducing compromised bone quality and OP.

Treg activity is influenced by both vitamin D and IL-33. Normal tissue repair, as well as bone and skin homeostasis, requires both vitamin D and IL-33 to locally expand Treg cells. The impact of Foxp3+ Treg cell derangement is involved in the autoimmune inflammatory processes of Pso (125). IL-33 promotes the recruitment of Treg cells into the site of injury, where they suppress inflammation. Through Treg recruitment and inhibition of NF-kB mediated gene transcription, IL-33 exerts protective effects on bone. Also, vitamin D may exert its immune regulatory properties through the induction of Treg cells. Low levels of vitamin D in Pso patients could dysregulate immunological homeostasis by decreasing the number of circulatory Tregs (46, 80). Vitamin D modulates CD4+ T cell function, reversing the defective induction of IL-10-secreting regulatory T cells. IL-33 induces IL-10-producing regulatory B cells and promotes IL-10 production in macrophages (125, 126).

### PTH Regulation by Vitamin D and IL-33

The links between vitamin D and IL-33 are both hormonal and immunological. PTH, the main hormone that regulates bone turnover and whose production is dependent on the levels of calcium and vitamin D in the body, is, in turn, strictly dependent on the IL-33/ST2 axis (127). Calcium metabolism is finely regulated by vitamin D and IL-33 in concert with PTH. The latter elevates calcium levels and reduces phosphorus levels in the blood. Calcium is a major regulator of sequential keratinocyte differentiation through the different layers of the epidermis until the stratum corneum is formed and is involved in signaling pathways that are central to desmosome and tight junction formation. Calcium receptors initiate the intracellular signaling cascade, driving differentiation in response to extracellular calcium. Calcium metabolism is also central to bone turnover. It is an essential component of hydroxyapatite crystals which confer hardness and resistance to the skeleton through deposits in the extracellular bone matrix. Vitamin D deficiency involves modification of calcium–phosphorus metabolism and increased secretion of PTH, which leads to increases in bone resorption and matrix demineralization. However, PTH could also function as an important osteoanabolic factor when administered pharmacologically (120, 128).

IL-33 and its receptors have roles in the PTH control of bone turnover (129). IL-33 mRNA levels in osteoblasts are increased by PTH and M oncostatin (127), and a positive correlation between PTH and IL-33 serum levels in postmenopausal OP has been observed (99). IL-33 production is stimulated by PTH, which contributes to the osteoanabolic effects of such a hormone. Vitamin D deficiency increases PTH secretion (78). As a consequence, IL-33 also increases, thus modulating the effects of PTH on bone remodeling (127). Therefore, the cytokine IL-33 represents a target of PTH and, synergistically with vitamin D, increases bone matrix mineralization by osteoblasts. IL-33 induced expression of the RANKL-encoding gene has been demonstrated in osteoblasts. RANKL, the major osteoclastogenic cytokine, is produced as a transmembrane protein, whose proteolytic processing is promoted by PTH, leading to the release of its soluble form. RANKL secretion, therefore, depends on integrated actions of both IL-33 and PTH (130). Moreover, both vitamin D and PTH control the levels of sST2. Serum sST2 correlates positively with serum phosphorus and negatively with serum calcium. The osteocyte-derived factor FGF-23, which inhibits PTH and promotes renal phosphorus excretion, also antagonizes some vitamin D effects. The IL-33/ST2 axis is, therefore, a PTH target that is able to both promote osteoblastic calcium deposition in the bone matrix and inhibit osteoclastogenesis (36). Increased levels of PTH induce elevation of sST2 (129). These data suggest that sST2 may play important roles in bone metabolism disorders, such as OP, as well as in inflammatory diseases, such as Pso.

Box 4 **Vitamin D/IL-33 crosstalk.**

Key points:

1. *Shared immunoregulatory and homeostatic effects:*

Promotion of Th2 lymphocyte differentiation

Induction of immunoregulatory cells

Downregulation of inflammatory cytokine and chemokine production

Regulation of bone remodeling process

Modulation of PTH control of bone turnover

2. *Contrasting roles:*

Protective role of vitamin D in allergic diseases vs the induction of allergic inflammation by IL-33

Enhanced inflammatory osteoclastogenesis by IL-33-induced secretion of IL-31 vs bone protection by vitamin D

IL-33 induced and amplified inflammatory circuits vs vitamin D anti-inflammatory activity in Pso

## The Paradox of the Vitamin D and IL-33 Relationship in Pso-Associated OP

Increased bone resorption with consequent appearance of OP is commonly associated with Pso and, in particular, with the more evolutionary forms characterized by systemic inflammation and joint involvement. It has been ascertained that vitamin D exerts protective effects in both Pso and OP (9). On the contrary, IL-33 is considered a pathogenetic cytokine in Pso, whereas its effects on bone are variable. Therefore, the role of IL-33 seems to be contrasting (111). In reality, this apparent contradiction could be explained by the multiplicity of functions of this cytokine which, depending on the type of tissue, the immune environment, and the presence of other associated factors, can have different effects. It could be hypothesized that, notwithstanding increased IL-33 levels, the skeleton is instead more sensitive to a wide range of osteoporotic risk factors that are increased during Pso, including inflammation and vitamin D deficiency, which potentiate each other. Furthermore, most studies have shown compartmentalization of IL-33 in Pso with increased concentrations in skin lesions but not in the serum (114), potentially explaining the lack of protective effects on bone. The final effect of the IL-33/ST2 axis in both Pso and OP therefore depends on the reciprocal relationship between its various components which influence each other through complex regulatory mechanisms and positive and negative feedback circuits (113). For example, the effect of an increase in IL-33 could be counterbalanced by a consensual increase in its decoy receptor sST2 or, on the contrary, it could be enhanced by increased expression of its receptor on different cell types (66). In turn, there are several types of IL-33 target cells, including immune cells, mesenchymal stromal cells, and epithelial cells (85). Therefore, depending on the target cell type, the effect of the cytokine could vary from pro-inflammatory to anti-inflammatory. In the immunopathogenetic processes driving inflammation, the co-operation of IL-33 with IL-17, IL-22, TNF-alpha, IFN-gamma, or other inflammatory factors has been suggested. In this way, the proinflammatory functions of IL-33 could prevail on its immunoregulatory properties, as observed in several autoimmune diseases, including Pso (129).

The bone protective effect of IL-33 could be masked or prevented by concomitant factors characterizing Pso, such as the deficiency of vitamin D and the consequently altered PTH-mediated calcium–phosphorus metabolism, the prevalence of Th1/Th17 systemic inflammation with an increase in osteoclastogenic cytokines, and mechanisms of counter-regulation of IL-33 signaling associated with the inflammatory process (99). On the other hand, IL-33 itself can perform different and contrasting functions: it can act as a pro-inflammatory factor in some pathological conditions but also as an alarmin with protective functions in response to danger signals, cell injury, and tissue damage (37).

Both vitamin D deficiency and increased circulating sST2 decoy receptor in Pso negatively impact the bone, favoring the production of Th1 rather than Th2 cytokines, suppressing the development of Treg cells and the production of regulatory and anti-inflammatory cytokines, thus promoting osteoclastogenesis and bone resorption (88, 94). The frequent of onset of OP during Pso could also be conditioned by the effective variability of effects of IL-33 on bone remodeling, which are dependent on a wide range of other factors (inflammatory microenvironment, influence of other cytokines, hormones, and vitamins) (9). A vitamin D deficiency could somehow nullify the protective effect of IL-33 on bone through mechanisms that are still unclear (121).

**Figure 1:**
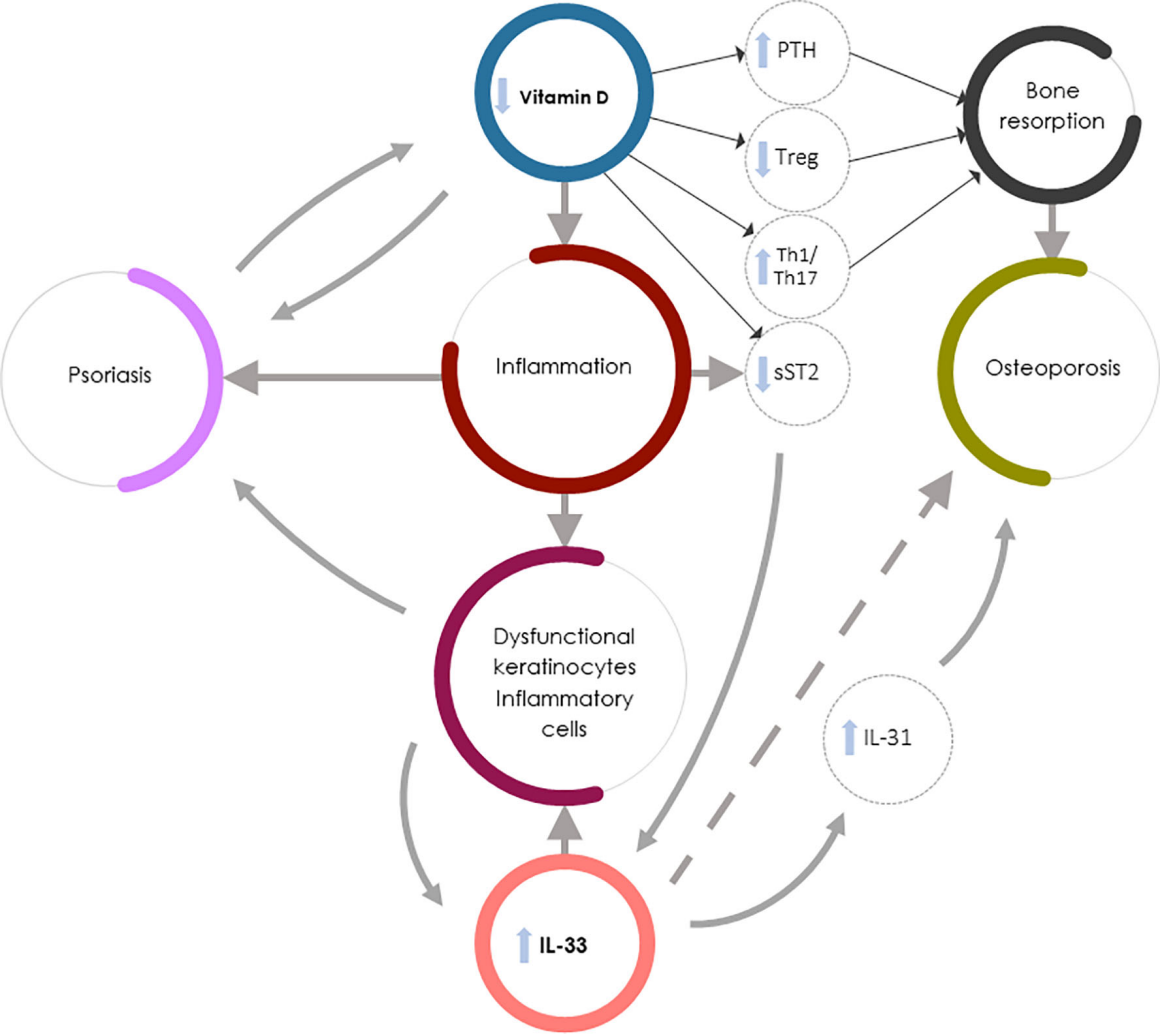
Vitamin D deficiency and increased IL-33/ST2 expression in Pso-associated OP. IL-33 could exert contrasting roles in Pso and OP: it might drive the inflammation underlying Pso by inducing the production of dysfunctional keratinocytes and inflammatory cells but could exert protective effects in OP (dashed arrow). Vitamin D, in synergy with IL-33, regulates bone metabolism through PTH release and function, but also increases the soluble decoy receptor sST2 production, thus regulating IL-33 function. The direct action of vitamin D in inhibiting IL-33 function on bone cells is, however, less important than other vitamin D-mediated osteoprotective mechanisms (e.g., the vitamin D capacity to both inhibit Th1 and Th17 inflammation and induce bone protective Th2-type responses). In Pso-associated OP the complex interaction between vitamin D deficiency and increased IL-33/ST2 expression therefore leads to osteoclastogenesis and bone resorption through several mechanisms: PTH hyperproduction, impaired Treg function, increased Th1 and Th17 inflammatory and osteoclastogenic cytokine production, and decreased sST2 expression by lymphocytes and epithelial cells, resulting in an increase in IL-33 induced skin inflammation. Furthermore, the increase in IL-33 in psoriatic patients leads to an increased production of IL-31 which contributes to the worsening of bone loss.

**Figure 1** summarizes the complex interaction between vitamin D deficiency and increased IL-33/ST2 axis expression leading to increased bone resorption and OP in Pso.

## Conclusions

In summary, there is clinical evidence that Pso, especially if associated with arthritis and a more advanced age, is associated with hypovitaminosis D, inflammation, and OP, and these factors might shift the effect of IL-33 from osteoprotective to proinflammatory and osteoclastogenic (39). The IL-33 levels in subjects with Pso reflect of increased inflammation, driving OP development. Different hypotheses could explain this paradox. For example, it has been recently demonstrated that the production of the soluble decoy receptor sST2 is enhanced by vitamin D (94). Since sST2 neutralizes the effect of IL-33, it is considered an anti-inflammatory factor in conditions in which IL-33 takes place in the driving inflammatory processes, such as asthma and Pso, and IL-33 neutralization may represent a novel therapeutic approach in these diseases. On the contrary, in the skeleton, the role of IL-33 is likely protective against OP and its sST2 mediated neutralization is detrimental (66). Therefore, in Pso-associated OP, the final effect of vitamin D deficiency and IL-33/ST2 axis overexpression is overall increased bone resorption due to the prevalence of proinflammatory and dysmetabolic processes (9, 66).

## Author Contributions

All authors contributed to the article and approved the submitted version.

## Conflict of Interest

The authors declare that the research was conducted in the absence of any commercial or financial relationships that could be construed as a potential conflict of interest.
